# The prevalence and outcome of effusive constrictive pericarditis: a systematic review of the literature

**DOI:** 10.5830/CVJA-2011-072

**Published:** 2012-06

**Authors:** Mpiko Ntsekhe, Patrick J Commerford, Bongani M Mayosi, Charles Shey Wiysonge

**Affiliations:** Cardiac Clinic, Department of Medicine, Groote Schuur Hospital and University of Cape Town, Cape Town, South Africa; Cardiac Clinic, Department of Medicine, Groote Schuur Hospital and University of Cape Town, Cape Town, South Africa; Cardiac Clinic, Department of Medicine, Groote Schuur Hospital and University of Cape Town, Cape Town, South Africa; Institute of Infectious Disease and Molecular Medicine, and School of Child and Adolescent Health, University of Cape Town, Cape Town, South Africa

**Keywords:** effusive constrictive pericarditis, prevalence, pericardiectomy and death

## Abstract

**Abstract:**

There is sparse information on the epidemiology of effusive constrictive pericarditis (ECP). The objective of this article was to review and summarise the literature on the prevalence and outcome of ECP, and identify gaps for further research. The prevalence of ECP ranged from 2.4 to 14.8%, with a weighted average of 4.5% [95% confidence interval (CI) 2.2–7.5%]. Sixty-five per cent (95% CI: 43–82%) of patients required pericardiectomy regardless of the aetiology. The combined death rate across the studies was 22% (95% CI: 4–50%). The prevalence of ECP is low in non-tuberculous pericarditis, while pericardiectomy rates are high and mortality is variable. In this review, of 10 patients identified with tuberculous ECP, only one presumed case had a definite diagnosis of ECP. Appropriate studies are needed to determine the epidemiology of ECP in tuberculous pericarditis, which is one of the leading causes of pericardial disease in the world.

Effusive constrictive pericarditis (ECP) is a clinical haemodynamic syndrome in which constriction of the heart by the visceral pericardium occurs in the presence of a compressive pericardial effusion. ECP is believed to be a rare manifestation of pericardial disease^1^ that occurs as part of a continuum from effusive to constrictive pericarditis. The outcome of ECP with regard to the development of constrictive pericarditis, pericardiectomy rates and death is not well defined.^2^ In the only prospective study of ECP, the prevalence was 6.8% of patients undergoing pericardiocentesis and 1.2% of all patients referred with effusive pericarditis.^3^ In the same study, 46.7% of participants with the diagnosis underwent pericardiectomy within four months, and the overall mortality rate was 60% over the subsequent seven-year mean follow-up period.^3^

The influence of the aetiology of pericarditis on the prevalence and outcome of ECP is not known. For example, tuberculous pericarditis is associated with significant inflammation,^4^ chronicity,^5^ and a high rate of development of constrictive pericarditis in about 25% of cases.^5^-^7^ It is likely therefore that the prevalence of ECP in patients with tuberculous pericarditis may be much higher than seen in acute forms of pericardial disease, such as idiopathic or viral pericarditis, which have formed the basis of the previous studies of ECP.^8^

With regard to the natural history, in the study of Sagrista-Sauleda, those with neoplastic disease had a high mortality and low pericardiectomy rate, whereas those with idiopathic disease had a low mortality rate but high pericardiectomy rate.^3^ The impact of the aetiology of pericarditis on these outcomes of ECP among patients whose life expectancy is not severely limited by malignant disease is not known.

There are very few investigators who have used the ‘gold standard’ to establish the diagnosis of ECP, which is invasive measurement of intra-pericardial and intra-cardiac pressures before and after pericardiocentesis.^2^ Even though non-invasive tools, such as echocardiography and magnetic resonance imaging are gaining wider acceptance as methods for establishing the diagnosis,^9^ none has been compared to invasive haemodynamic diagnosis of ECP.^9^,^10^

It has been proposed that visceral pericardiectomy may be necessary for a good clinical result in cases with ECP because drainage of pericardial fluid alone leads to incomplete relief of cardiac compression.^3^ The timely recognition of ECP therefore enables the clinician to choose the most appropriate therapy. Information about the prevalence and outcome of ECP is particularly important in the developing world, where tuberculosis causes hundreds of thousands of cases of pericarditis every year.^5^ There are at present no recommendations on the diagnosis and management of ECP in tuberculous pericarditis.

We have conducted a systematic review of the literature to determine the prevalence and outcome of ECP in patients with viral, tuberculous, uraemic, purulent and idiopathic pericarditis. The outcomes of interest were pericardiectomy and mortality rates at 12 months. Furthermore, we determined whether the prevalence and the outcome of ECP were related to the aetiology of the effusion. We limited the review to observational studies of pericarditis due to these non-neoplastic medical conditions that commonly progress to constrictive pericarditis.^1^

## Methods

MEDLINE, EMBASE and Google Scholar were searched for English-language publications of observational studies of ECP that were conducted from inception of the respective database through to December 2009. Search terms included: acute pericarditis, pericardial effusion, ECP, pericardial tamponade, cardiac tamponade, tuberculous pericarditis, uraemic pericarditis, purulent pericarditis, idiopathic pericarditis, viral pericarditis and constrictive pericarditis. Limits included: the English language, human beings and the following MeSH terms (‘Case-Control Studies’[MeSH] OR ‘Cohort Studies’[MeSH] OR ‘Epidemiologic Studies’[MeSH] OR ‘Cross-Sectional Studies’[MeSH] OR ‘Retrospective Studies’[MeSH] OR ‘Prospective Studies’[MeSH]). In addition to searching the databases, we contacted researchers in the field, and searched the bibliographies of published reviews and studies on pericardial disease for relevant studies.

The eligibility criteria for inclusion and exclusion from the study, which are based on the Loney criteria for critical appraisal of research articles on prevalence of disease, are shown in Table 1.^11^ To be included in the review, a study had to provide sufficient information to enable determination of the proportion of study participants diagnosed with ECP and at least six other eligibility criteria.

**Table 1.** Eligibility Criteria For Studies Of The Systematic Review

*Inclusion criteria*
1. The study design was observational (case control, cross sectional and cohort); cross sectional studies were accepted for the determination of prevalence.
2. A definition of the syndrome of effusive constrictive pericarditis was given.
3. The inclusion and exclusion criteria for the participants were clearly stated.
4. There was a clear description of the number of participants in the study.
5. The number or proportion of participants in the study with effusive constrictive pericarditis was clearly stated.
6. The method of diagnosis of effusive constrictive pericarditis was described and determined in an unbiased manner.
7. There was an adequate description of the study setting.
8. There was an adequate description of the study population.
*Exclusion criteria*
1. The number or proportion of participants with effusive constrictive pericarditis was not available.
2. The aetiology of pericarditis was a malignancy, myocardial infarction, aortic dissection, or trauma to the thorax.
3. The diagnosis of effusive constrictive pericarditis was based on clinical assessment only.

Studies where malignancy was the predominant cause of pericarditis were excluded from this systematic review because patients with this diagnosis generally do not survive long enough to develop constrictive pericarditis.^1^,^12^ Studies of patients with pericardial effusion that resulted from aortic dissection, myocardial infarction, and trauma to the thorax were also excluded because pericardial sequelae are uncommon among long-term survivors of these conditions.^1^,^13^-^15^

After the relevant studies were selected, individual patient data were extracted and reviewed in order to exclude patients with malignancy-associated ECP. Where relevant data could not be extracted from the published manuscripts, we were able to obtain the information on individual participants from the study authors. We conducted a meta-analysis of the individual patient data using the StatsDirect software (www.statsdirect.com). For the meta-analysis, StatsDirect first transformed proportions into a quantity (the Freeman-Tukey variant of the arcsine square root-transformed proportion) suitable for the usual fixed and random-effects summaries.^16^,^17^ The pooled prevalence was calculated as the back-transform of the weighted mean of the transformed proportions, using inverse arcsine variance weights for the fixed-effects model^16^ and DerSimonian-Laird^17^ weights for the random-effects model.

We used the Cochran Q test to assess statistical heterogeneity between studies and, in the absence of significant heterogeneity (*p* > 0.1), combined the data using a fixed-effects method. Otherwise, we used the random-effects method. In addition, we used Higgins *I*^2^ statistic to quantify inconsistency across the studies included in the meta-analysis. The test statistic describes the percentage of the variability in effect it estimates that is due to true heterogeneity rather than chance. The closer the *I*^2^ value is to 100%, the more likely it is that true heterogeneity exists, and therefore the less reliable the combined estimate becomes.

MN conducted the electronic searches and selected the studies, all of which were reviewed by CW and BMM. The reporting of the systematic review is in keeping with standard recommendations for reporting systematic reviews of observational studies.^18^

## Definitions

Effusive constrictive pericarditis was classified as definite or probable, based on the methods used to establish the diagnosis.^2^,^9^ Studies where the diagnosis was based on clinical assessment alone were rejected.

Patients were classified as having definite ECP if the diagnosis was based on intra-pericardial and intra-cardiac haemodynamics, determined before and after pericardiocentesis. This haemodynamic definition required that: (1) the pre-pericardiocentesis transmural filling pressure (i.e. the difference between the elevated intra-pericardial pressure and the right atrial pressure) was less than 2 mmHg; (2) the post-pericardiocentesis intra-pericardial pressure fell to near 0 mmHg; and (3) the post-pericardiocentesis right atrial pressure failed to fall by 50% or to a level below 10 mmHg.^3^

The diagnosis of ECP was considered probable if it was established on the basis of echocardiography or magnetic resonance imaging. There are no published prospectively derived consensus diagnostic criteria for ECP using these imaging modalities,^9^ but widely accepted criteria include evidence of the following criteria in a patient with a pericardial effusion: (1) pericardial thickening; (2) abnormal or paradoxical movement of the interventricular septum; (3) a plethoric dilated inferior vena cava with reduced narrowing during inspiration; and (4) marked respiratory variation of the mitral inflow Doppler pattern. Finally, the diagnosis of ECP was rejected if it was established without ancillary imaging or haemodynamic assessment, i.e. if the diagnosis was made on clinical assessment alone.

## Results

**Fig. 1.**
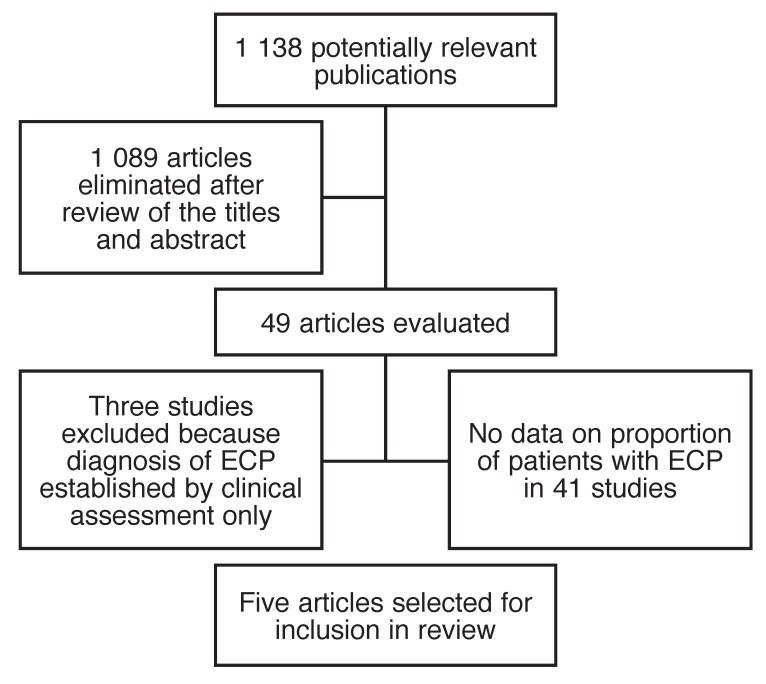
Flow chart for selection process.

A flow chart for the selection process is provided in Fig. 1. Five studies were included in the systematic review.^3^,^19^-^22^ The five studies had a total of 642 patients, 26 of whom met diagnostic criteria for ECP; 58% (15/26) had probable ECP and 42% (11/26) definite ECP. Of the 26 patients, 50% (13/26) had idiopathic pericarditis, 38% (10/26) had tuberculous pericarditis, 8% (2/26) had post-radiation pericarditis and 4% (1/26) post-pericardiotomy pericarditis.

## Prevalence of effusive constrictive pericarditis

The study design and strength of diagnosis of ECP varied across the five selected studies. Three of the five studies were prospective cohorts.^3^,^19^,^21^ One of the three prospective case series was a single-centre South African study, designed to determine the 30-day and one-year outcomes of consecutive patients with predominantly tuberculous pericarditis, who were each given a standardised therapeutic protocol, which included pericardiocentesis.^19^ The proportion of those with ECP was 2.6% based on clinical and echocardiographic criteria.

The second prospective case series was a single-centre French study designed to determine the role of surgical pericardioscopy as a diagnostic tool among patients with large pericardial effusion of uncertain aetiology.^21^ The proportion of patients diagnosed with ECP was reported as 1.4%. All patients underwent pericardiocentesis, and echocardiography was used to assess pericardial physiology and content.

The third prospective case series was a single-centre Spanish study, which aimed to determine the prevalence of ECP and the incidence of pericarditis-related outcomes over a median follow-up period of seven years.^3^ Consecutive participants presenting with a diagnosis of pericardial tamponade over 15 years underwent measurement of the pre- and post-pericardiocentesis intra-pericardial and right atrial pressures. The prevalence of ECP was 5.8% in those patients undergoing pericardiocentesis, 6.8% in those with clinical tamponade, and 0.93% in patients with any pericardial disease.^3^

The remaining two studies of patients with a probable diagnosis of ECP were designed to (1) determine the long-term outcome of patients with symptomatic effusion;^22^ and (2) compare echocardiographic differences between tuberculous and idiopathic pericardial effusions.^20^ The prevalence of ECP in these two studies was 4.3 and 14.8%, respectively.

**Fig. 2.**
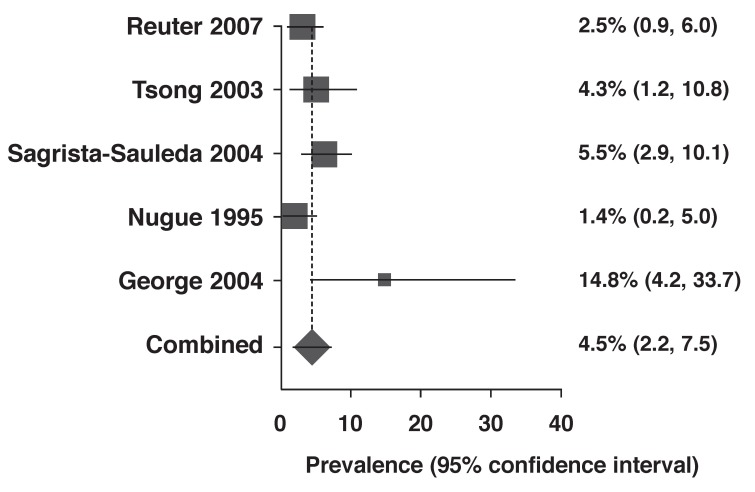
Forest plot for the prevalence of ECP (random effects).

Overall there was significant variability in the prevalence of ECP across the five studies (*p* = 0.04; *I*^2^ = 61%); therefore we used both the random-effect and fixed-effect meta-analysis models to combine the prevalence. Using the fixed-effect model, the pooled prevalence of ECP in the five studies was 4.0% (95% CI: 2.7–5.7%). This increased marginally to 4.5% (95% CI: 2.2–7.5%) using the random-effects model (Fig. 2).

## Outcomes of patients with effusive constrictive pericarditis

One-year mortality data was available for only nine participants with non-malignant disease from two studies.^19^,^22^ These mortality rates are provided in Table 2.

**Table 2.** 12-Month Mortality And Pericardiectomy Rates Of Participants With Non-Neoplastic Effusive Constrictive Pericarditis

*Study*	*Absolute number of study participants with non-neoplastic ECP*	*Number of patients with ECP who underwent pericardiectomy within 12 months*	*Number of patients with ECP dead at 12 months*
Sagrista-Sauleda 2004	11	7/11 (64%)	Mortality data at 12 months not available for all patients
Reuter 2007	5	2/5 (40%)	2/5 (40%)
Tsang 2003	4	4/4 (100%)	0/4 (0%)
Nugue 1996	2	Pericardiectomy data not available	Mortality data not available
George 2004	4	Pericardiectomy data not available	Mortality data not available
Total	26	13/20 (65%)	2/9 (22%)

Two of the nine patients were dead at 12 months; one from peri-operative complications, and the other with tuberculous ECP died while awaiting pericardiectomy. The combined death rate across the studies was 22%, with wide 95% confidence intervals (4–50%) due to the small numbers involved. Seven patients did not undergo pericardiectomy. These seven included: the patient with tuberculosis who died from heart failure while awaiting surgery, three participants, also with tuberculosis, who did not consent to the procedure, and three participants with idiopathic disease in whom a conservative ‘wait-and-see’ approach had been adopted. The six participants, who survived the early stages of their illness without surgery were alive and well at their last follow-up visit.

Only three of the studies provided data on the pericardiectomy rates.^3^,^19^,^22^ Overall, the combined pericardiectomy rate was 65% (95% CI: 43–82%) and the between-study variability in pericardiectomy rates was marginally significant (*p* = 0.10; *I*^2^ = 56%). A breakdown of the pericardiectomy rates by aetiology revealed that 73% of participants with idiopathic ECP, 60% of those with tuberculous ECP, and 50% of those with ECP of other aetiologies underwent the pericardiectomy.

The persistence of heart failure was the reason for surgery in 54% of cases, making it the most common indication, followed by prophylaxis against progression to fibrous constrictive pericarditis in 23%. Recurrence of pericardial effusion was an indication in 15%. In only 8% was the operation performed because of progression to non-effusive fibrous constrictive pericarditis.

## Discussion

This systematic review highlights that there are very few prospective studies on the prevalence and outcome of ECP. The prevalence of this syndrome in the available studies ranged from 1.4 to 14%. Although there was little information to ascertain the mortality rate reliably, the pericardiectomy rate was clearly high (44–100%).

There was a total of 10 participants who had effusive constrictive tuberculous pericarditis in this review, one of whom had a definite diagnosis of ECP. Commerford and Strang have suggested that ECP may be a common form of presentation of tuberculous pericarditis that frequently progresses to fibrous constrictive pericarditis.^8^ By contrast, the IMPI Africa Registry has suggested that using clinical criteria alone, ECP may be present in only 15% of cases of tuberculous pericarditis.^23^

The results of this comprehensive review show a low prevalence of ECP in patients with tuberculous pericarditis, which ranged from 3 to 14%. It is noteworthy that there are no studies that have systematically used an invasive haemodynamic method to establish the diagnosis of effusive constrictive disease in patients with tuberculous pericarditis. There is therefore a need for a definitive study of the prevalence of tuberculous ECP that is based on invasive haemodynamic methods.

Although the pericardiectomy rate across the studies was high, the indications for surgical intervention were not uniform among the 13 participants who had the operation. A significant proportion of patients who were managed conservatively had complete resolution of their effusive constrictive disease. This suggests that there is room for a study to test a strategy of watchful waiting compared to prophylactic pericardiectomy in those without persistence of heart failure.

Finally, the mortality rate for tuberculous pericarditis in the HIV era is as high as 40% in patients with AIDS, at the end of six months of treatment with anti-tuberculosis medication.^24^ Despite the absence of data on mortality in patients with non-neoplastic ECP, it is possible that because of its well-documented haemodynamic sequelae,^2^ the pericardial syndrome is associated with a higher mortality rate than those without the syndrome.

## Conclusion

In light of the lack of clarity on the prevalence of ECP among patients with proven tuberculous pericarditis, the role of prophylactic pericardiectomy in cases of varying aetiology, and the impact of the syndrome on mortality, a study of well-characterised participants with adequate follow up and clearly defined outcomes is required to inform the development of clinical guidelines on the diagnosis and management of effusive constrictive pericardial disease.
